# Development and preliminary validation of the 'Caring for Country' questionnaire: measurement of an Indigenous Australian health determinant

**DOI:** 10.1186/1475-9276-7-26

**Published:** 2008-12-18

**Authors:** Christopher P Burgess, Helen L Berry, Wendy Gunthorpe, Ross S Bailie

**Affiliations:** 1 Menzies School of Health Research, Institute of Advanced Studies, Charles Darwin University, Darwin, NT, Australia; 2 National Centre for Epidemiology & Population Health, The Australian National University, Canberra, ACT, Australia

## Abstract

**Background:**

'Caring for Country' is defined as Indigenous participation in interrelated activities with the objective of promoting ecological and human health. Ecological services on Indigenous-owned lands are belatedly attracting some institutional investment. However, the health outcomes associated with Indigenous participation in 'caring for country' activities have never been investigated. The aims of this study were to pilot and validate a questionnaire measuring caring for country as an Indigenous health determinant and to relate it to an external reference, obesity.

**Methods:**

Purposively sampled participants were 301 Indigenous adults aged 15 to 54 years, recruited during a cross-sectional program of preventive health checks in a remote Australian community. Questionnaire validation was undertaken with psychometric tests of internal consistency, reliability, exploratory factor analysis and confirmatory one-factor congeneric modelling. Accurate item weightings were derived from the model and used to create a single weighted composite score for caring for country. Multiple linear regression modelling was used to test associations between the caring for country score and body mass index adjusting for socio-demographic factors and health behaviours.

**Results:**

The questionnaire demonstrated adequate internal consistency, test-retest validity and proxy-respondent validity. Exploratory factor analysis of the 'caring for country' items produced a single factor solution that was confirmed via one-factor congeneric modelling. A significant and substantial association between greater participation in caring for country activities and lower body mass index was demonstrated. Adjusting for socio-demographic factors and health behaviours, an inter-quartile range rise in caring for country scores was associated with 6.1 Kg and 5.3 Kg less body weight for non-pregnant women and men respectively.

**Conclusion:**

This study indicates preliminary support for the validity of the caring for country concept and a questionnaire designed to measure it. This study also highlights the importance of investigating Indigenous-asserted health promotion activities. Further studies in similar populations are merited to test the generalisability of this questionnaire and to explore associations with other important Indigenous health outcomes.

## Background

In Australia's Northern Territory (NT) more than 70% of the Indigenous population live on the 49% of the landmass and 85% of the coastline that is Indigenous-owned [1,2]. Colonial contact has largely displaced Indigenous peoples from their ancestral estates [3], relocating populations to remote area townships on Indigenous-owned lands [4]. This policy of centralisation, pursued in the last decade with increasing vigour under the rubric of 'mainstreaming' [5,6], runs counter to evidence suggesting negative health outcomes for these peoples [7,8]. In the words of an Indigenous Australian:

"Our identity as human beings remains tied to our land, to our cultural practices, our systems of authority and social control, our intellectual traditions, our concepts of spirituality, and to our systems of resource ownership and exchange. Destroy this relationship and you damage – sometimes irrevocably – individual human beings and their health" [9].

Remote Indigenous townships are often described as chaotic and dysfunctional settings marked by social pathologies [10,11] and pervasive socio-economic disadvantages [12]. Consistent with their extreme disadvantage, Indigenous Australians' life expectancy is 17 years less than the Australian average with mortality rates for those aged 35–54 more than five times higher than the national average [10]. This also compares poorly with the life expectancy for Indigenous populations in New Zealand, Canada and the United States [13]. For Indigenous Australians in the NT, a disproportionate burden of disease linked to inactivity, malnutrition, and tobacco dependence underpins this wide health disparity [14]. Non-insulin dependent diabetes mellitus (NIDDM) and cardiovascular disease account for 40% of excess Indigenous mortality and over 21,800 preventable hospital admissions annually [10]. Mainstream health promotion campaigns have been ineffective in decreasing this burden of disease in such challenging circumstances.

Australia's peak health research body, the National Health and Medical Research Council (NHMRC), recognises that much previous health research "has not contributed in a significant or systematic way to improved health outcomes for Aboriginal and Torres Strait Islander populations" [15]. Indigenous critics have demanded a shift in research towards identifying 'what works', including (i) improving the social determinants of health, (ii) identification of cultural drivers of resilience and health gains and (iii) the stipulation that solutions may arise from outside the health domain [15].

There is a clear and urgent need for effective Indigenous health interventions. Indigenous Australians assert that their relationship to ancestral land and sea is a prerequisite for health [3]. This relationship is poorly understood, unmeasured and receives only tacit recognition in Australia's National Strategic Framework for Indigenous health [16].

### Healthy Country Healthy People

Country is an Indigenous vernacular term encompassing an interdependent relationship between Indigenous peoples and their ancestral estates.

"Country is multi-dimensional – it consists of people, animals, plants, Dreamings; underground, earth, soils, minerals and waters, air... People talk about country in the same way that they would talk about a person: they speak to country, sing to country, visit country, worry about country, feel sorry for country, and long for country" [17].

Country is considered sentient [18], rewarding those who labour to maintain its mythic and physical integrity with a bountiful harvest and bestowing physical, spiritual and social wellbeing [19]. Maintenance of health and well-being requires hard work, sustained through mutual care of kin, non-human affiliations and observance of ethical conduct described by the law or dreaming that is encoded within country [17,19-21]. Failure to observe these obligations may result in human sickness or ecological catastrophes [18,22].

Urbanisation of remote Indigenous populations constrains opportunities to fulfil customary obligations to country. Although absence of landowners contributes to ecological degradation [23], contemporary forms of natural resource management have emerged to tackle environmental issues and maintain links with ancestral estates [24]. Indigenous ranger programs undertake a broad array of activities, including border protection, quarantine, and essential ecological services [3], that overlap with customary obligations.

Indigenous Australians living in homelands, where caring for country practices are common [25,26], appear to have better health outcomes compared to centralised populations [7,8,27,28]. Similarly, reinvigoration of a 'traditional lifestyle' delivers significant health improvements, even for those with established NIDDM [29]. This is consistent with international examples of programs leveraging off extant cultural strengths to successfully combat substance abuse and chronic diseases [30,31]. In the international literature, however, there is a dearth of studies that explicitly engage, measure and validate Indigenous-asserted health constructs, potentially overlooking significant wellsprings of health promotion within Indigenous communities.

The absence of any measure of Indigenous engagement in caring for country activities limits the potential to evaluate or inform policy decisions based on associations with purported superior health outcomes [32].

### Aims of the present study

We aimed to (i) test the validity of a questionnaire measuring Indigenous participation in caring for country activities and (ii) investigate the association between caring for country and an external health reference, body mass index (BMI), which is associated with the development of NIDDM and cardiovascular disease [33,34]. We expected that higher levels of participation in caring for country activities would deliver more opportunities for exercise and a more nutritious diet and would thus be associated with a lower BMI.

## Methods

This study was initiated by a traditional land-owner from an Arnhem land community, who requested that researchers investigate the links between participation in natural resource management activities and human health. Ethics approval was obtained in 2004 from Charles Darwin University (H04053) and the NT Department of Health and Community Services (04/35) which includes an Aboriginal ethics sub-committee approval process. Approval was also granted from the Indigenous governed community health board and the Indigenous governed outstation resource organisation. The study setting was a large remote Indigenous community in Arnhem Land. The township, a conglomerate of 11 language groups established in 1957, is surrounded by 32 established homelands. This community has undergone a rapid transition over 50 years, becoming largely sedentary and reliant on income support. Within the population there is wide variation in caring for country participation.

### Participants and procedures

Participants volunteering for the community preventive health check program were 301 Indigenous adults (177 men, 124 women) aged 15 to 54 years (M = 30.96, S_x _= 10.15), comprising 23.4% of the community population in this age range [4]. The cohort age structure differed slightly, but not significantly, from the census profile (Pearson's Chi Square statistic: 10.04, *p *= .19) [4]. Of the participants, 298 (99%) completed an interviewer-administered caring for country questionnaire. The same interviewer administered the questionnaire on each occasion. Approximately one-third (N = 102) of participants came from 16 homeland communities and the remainder from the township. We undertook purposive sampling to recruit participants with different levels of involvement in formal and customary caring for country activities. Participants were from homelands, township residences, workplaces (rangers and non-rangers) and public spaces (outside the community store and community council buildings).

Three participants did not complete the caring for country questionnaire. Ten could not have weight and height recorded on the standardised equipment due to disability (N = 1) and equipment delays in the aftermath of tropical cyclone Ingrid (N = 9). Nine failed to complete questions on physical activity and diet. Five women were in the early stages of pregnancy and these participants were excluded from the final regression modelling of BMI. As there were fewer than 5% missing data for any variable, and no missing data for most variables, we imputed missing data using Full Information Maximum Likelihood estimation. Imputed means and standard deviations were identical or near-identical to those derived from the dataset with missing values; we used the imputed values for all further analyses.

Spending time on country, the seasonal burning of annual grasses, gathering of food and medicinal resources, performing ceremonies, production of artworks and protecting sacred areas are identifiable 'caring for country' activities [18,19,35]. Participants reported how often they participated in these six activities over the preceding twelve months on a four point ordinal response format: 1 = "Not much (none in the last year)"; 2 = "A little bit (a few days in the last year)"; 3 = "A fair bit (a few weeks in the last year)"; 4 = "Heaps (a few months in the last year)" (Additional file 1). Two further questions investigated time spent on homelands: (i) "In the last year, where did you spend most of your time living?" (the township name, homeland or other) and (ii) "How much time have you lived in a homeland/outstation in the last five years?" (all the time, a few months each year, a few weeks each year, a few days each year or none).

Follow-up and treatment were provided as clinically indicated, including a feedback letter outlining an individually tailored strategy for good health. At the time of feedback (a minimum of two weeks later) participants completed the questionnaire a second time with the same interviewer. Sixty-six participants (22% of the cohort) repeated the questionnaire within 6 weeks (M = 30.7 days, S_x _= 7.99).

A senior Indigenous member of the community with well-established community links across all language groups and knowledge of all the participants also completed the questionnaire for each respondent. This 'proxy respondent' had not been involved in the health check program and had no knowledge of participant health outcomes or responses to the questionnaire. We compared the proxy's response with those of respondents', an acceptable and validated method to verify health related behaviours in remote Indigenous settings [36].

### Measures

#### Caring for country questionnaire development

The questionnaire was developed in four stages over a two-year period of collaboration with an Indigenous community in Arnhem Land.

##### Stage 1: Scoping Study, Literature review, consultation and participant observation

Several databases covering a range of disciplines were searched for material on Indigenous caring for country and health. These included: APAIS, MEDLINE, PubMed, CINAHL, ATSI-health, Anthropological index online, ISI. Ethnographies, textbooks and conference proceedings covering Indigenous themes were included and helped to identify leading authors in this field, who were contacted by phone or email. Six field trips of up to 2 weeks duration enabled the first author to establish relationships with key Indigenous and non-Indigenous informants and undertake participant observation of both formal 'ranger' programs and informal, customary management practices. While no previously validated measures of caring for country were identified, five potential questionnaire items were identified from extant literature: time on country, burning, using country, protecting country and ceremony. (Povinelli, 1993, Rose, 1992, p106–7).

##### Stage 2: Content Validity assessment with non-Indigenous informants

Four male non-Indigenous informants were identified during the scoping study. All had lived and worked in remote Indigenous communities for over 20 years. Three of these informants were still resident in remote communities at the time of consultation. One was resident in Darwin but maintained active involvement in Indigenous ranger programs. Three of the non-Indigenous informants had a direct association with the research community, and the fourth had no direct association with the community but was resident in a remote coastal Aboriginal community.

A sixth scale item, production of artefacts, was suggested and several plain language cues for each item were volunteered, corresponding to colloquial expressions in the community. An additional item concerning the reciprocal nature of caring for country, specifically the energy and vitality that arose from participation. (Thomson, 1975), was suggested by one informant. Subdivision of ceremonial activity between funeral rites and other ceremonies was also suggested

##### Stage 3: Content Validity assessment with Indigenous informants

Five Indigenous informants, four from the research community and one from outside the community assessed the content validity of the questionnaire. The four community informants (3 male and one female) were a purposive sample. All aged in their fifth decade, they were employees of disparate community agencies. They were from four different language groups. The community male informants represented the Indigenous rangers, an executive from the outstation resource centre and a member of the community health board. The community female informant was an employee of the women's centre. All community informants were fluent in English and several local Indigenous languages, identified with landowning groups and had in their lifetimes lived for extensive periods of time in remote homelands. The final Indigenous male informant, aged in his fourth decade, was based in Darwin and had over ten years of experience facilitating formal Indigenous natural resource management programs in widespread locations across the NT.

All Indigenous informants readily understood the purpose of the questionnaire and did not volunteer any additional items. The item regarding energy arising from caring for country was considered to be real and important but too difficult to include in the linguistically diverse research setting and was excluded at this stage. Item specific cues and quantification cues were considered intelligible and appropriate. The need for an interviewer administered questionnaire was highlighted. Three Indigenous informants felt that the division of ceremonial activity between funeral rites and other ceremonies was an artificial one and these two items were combined into a single ceremony category.

##### Stage 4: Construct validity assessment through key informant interview

Finally, a semi-structured interview with an Indigenous male from the community was undertaken based on the caring for country questionnaire developed in the previous three stages. This key informant, aged in his fifth decade, had well developed links across all language groups through his employment as an Aboriginal mental health worker. He had spent extensive periods in both homelands and in employment with non-Indigenous agencies in the township setting. This discussion was recorded on a digital voice recorder, predominantly in English, at the choice of the interviewee, but supplemented with Indigenous language to convey key concepts. Translations, where necessary, were supplied by the principal informant and verified [37].

In this community, caring for country activities were qualitatively associated with an holistic health construct, *an-ngurrunga-wana*, a state of vitality of mind, body and soul, roughly translated as "he-soul-big" [37]. This construct forms the *a priori *hypothesis for measure development. We expected all scale items would load on a single latent factor, *an-ngurrunga-wana*.

Further construct validity assessment of the items within the questionnaire was also guided by Reid's [38] 'Body, Land and Spirit' domains – an interpretive framework of Yolngu health beliefs in north east Arnhem land. Time on country, using country and burning are linked in practice [39], and involve direct interaction with specific landscapes. These three items may pertain to the dimension of land. Ceremony and protecting country are linked to spiritual beliefs and practices to maintain the spiritual integrity of landscapes [22] and may pertain to the dimension of spirit. The production of artefacts: carvings, paintings, weavings and other decorative or utility items are concrete expressions of specific landscapes or ancestral knowledge [35,40]. Artefacts may thus pertain to body or the 'material embodiment' of land and spirit domains. (Additional file 2)

The interviewer also collected data on primary place of residence, education, income, diet, physical activity, alcohol consumption and smoking status. While we expected that participants engaging in higher levels of caring for country would come from homelands [25,26], we wished to control for residence in our analysis because (i) caring for country participants could come from the township; (ii) not all homelands residents care for country and (iii) homelands residents may have differing dietary and physical activity factors, based on their isolation, which could potentially confound caring for country in predicting BMI.

#### Socio-demographic factors

Income was divided into three ordinal categories: 1 = unemployment benefits (lowest income); 2 = Abstudy (Aboriginal education support payments), Community Development Employment Program, carer allowance, child support, receiving payments for artefact production (middle income); 3 = salaried positions (highest income). This last category was rare, including only 1.3% of respondents. Educational attainment was categorised as: 1: no formal education; 2: primary education; 3: lower secondary; 4: year ten; 5: year twelve; 6: post school qualification. Higher levels of education have been asserted to deliver better health outcomes in Indigenous populations [41].

Diet data were collected with standardised visual cues depicting commonly available foodstuffs that participants reported consuming: never; sometimes; most days; every day. Physical activity was assessed by a question adapted from the Australian longitudinal study on women's health: "How many times a week do you exercise enough to get short of breath or huff and puff?" [42]. This was accompanied by visual cues depicting sporting activity, hunting, digging and ceremonial dancing. Participants reported; none; one or two times; three or four times; more than four times. Smoking status was assessed by asking: "Do you smoke tobacco?" (yes/no).

Weight was recorded on digital scales to the nearest 100 g and height to the nearest centimetre, using a mounted stadiometer, following accepted techniques [43]. Participants wore light clothing and had bare feet. BMI was derived by dividing weight in kilograms by the square of the person's height in metres.

### Statistical methods

Descriptive statistics were computed for the cohort. Item endorsement, inter-item correlations, quadratic weighted kappa scores for test-retest and proxy response reliability were calculated. Exploratory factor analysis was appropriate for a preliminary investigation of the factor structure underlying the items. The dataset was appropriate for exploratory factor analysis [44]: the questionnaire items were theoretically related; the study was designed for factor analysis; the dataset was factorable (multiple inter-item correlations > .3); the sample size was adequate and; sampling statistics were acceptable (Kaiser-Meyer-Olkin statistic = .84, Bartlett's test of sphericity *p *< .0001). Maximum likelihood factoring with oblimin rotation were used to allow for skewed data and a correlated solution (should the solution contain more than one factor) respectively. Evaluation criteria were consistency with theory, factor loadings exceeding .45 and eigenvalues > 1.0.

One-factor congeneric modelling (see Berry [45]), a sub-set of structural equations modelling, was used to (i) test and refine the exploratory factor solution and (ii) generate a set of accurate item weightings for the creation of a weighted composite scale (Range = .76–3.06, M = 1.93, S_x _= .67). The model was fitted using an asymptotic distribution free algorithm to accommodate non-normal distributions in the data. Model fit was assessed by a holistic appraisal of the χ^2 ^statistic, critical ratios, and a selection of goodness of fit indices (absolute fit, incremental fit and parsimony indices).

Recent research has suggested that breadth of community participation across a range of important types of participation is more strongly linked to wellbeing than is the mean amount of participation, or very high levels of any particular type of participation [45]. We investigated whether this may be true of any association between participating in caring for country and health. In our regression analyses, we compared a single weighted composite score for caring for country, derived from the one-factor model, with an index of total number of types of caring for country in which respondents participated. The index was created by dichotomising item scores by mean split, assigning a value of 0 (non-participator – below the mean) and 1 (participator – at or above the mean), and summing these scores. This generated a seven-point index (Range = 0–6, M = 2.73, S_x _= 2.22). An un-weighted composite scale and the index demonstrated satisfactory internal coherence (Cronbach alpha scores = .88 and .85 respectively). Cronbach alpha scores cannot be derived for weighted composites.

Multivariate logistic and ordinal logistic regression analyses were performed to evaluate whether caring for country predicted obesity-related health behaviours, controlling for socio-demographic factors and other health behaviours. Multiple hierarchical regression models were used to test the relationship between caring for country and BMI. The models included variables tapping social determinants, residence, health behaviours and caring for country. Analyses were performed using Stata [46], SPSS [47], and AMOS (structural equation modelling) [48].

## Results

### Weight

BMI was calculated for 301 participants (Range = 12.6–42.3, M = 22.91, S_x _= 5.58). On average, this was a lean population with mean BMI decreasing from 23.57 in those with the lowest level of participation in caring for country activities to 22.01 for those with the greatest level of participation (Table 1). Men were slightly leaner (BMI M = 22.47, S_x _= 4.92) than were non-pregnant women (BMI M = 23.67, S_x _= 6.43).

**Table 1:** Cohort characteristics by low, medium and high caring for country scores.

	Low (score: 6–12)		Medium (score: 13–18)		High (score: 19–24)	
	Mean	S_x_	Mean	S_x_	Mean	S_x_
Number of participants	122	N/a	105	N/a	74	N/a
% Male	61%	N/a	58%	N/a	57%	N/a
Age in years	29.27	10.50	30.84^	10.05	33.79	9.21
*Socio-demographics*						
% Resident in homelands	2.5%^^^	N/a	38.1%^^^	N/a	79.7%	N/a
Mean income level ^1^	1.50^^^	.52	1.90	.38	1.92	.27
Mean education level ^2^	3.03	1.05	2.89	.89	2.67	.83
*Health behaviours*						
% Smoker	70%	N/a	75%	N/a	74%	N/a
% Drinks alcohol	30.3%	N/a	37%^	N/a	20.3%	N/a
Physical activity ^3^	2.84^	.92	3.08^^^	.84	3.5	.61
Takeaway ^4^	2.26 ***	.66	1.97	.57	1.91	.52
Store Fruit ^4^	2.40	.74	2.51*	.78	2.25	.54
Store Vegetable ^4^	2.37	.71	2.57**	.77	2.25	.58
Bush meat ^4^	3.1^^^	.76	3.52^^^	.74	3.86	.37
Bush fruit and vegetable ^4^	2.71^^^	.92	3.27^^	.86	3.61	.64
*Health outcome*						
Body Mass Index	23.57	6.30	22.86	5.11	22.01	4.86

Notes: 1: 1 = lowest income, 2 = medium income, 3 = highest income

2: 1 = no formal education, 2 = primary school, 3 = lower secondary, 4 = year ten, 5 = year twelve, 6 = post school qualification

3: 1 = none, 2 = one or

two times a week, 3 = three or four time a week, 4 = more than four times a week 4: 1 = never, 2 = sometimes, 3 = most days, 4 = every day

*p < .05, **p < .01, ***p < .001: score is significantly *higher *than that of the next group

^p < .05, ^^*p *< .01, ^^^p < .001: score is significantly *lower *than that of the next group

### Caring for country

Quantification category endorsement of the caring for country items varied from 3.7% to 66.4%. Item response skew was uncommon except for the artefact production item. Pearson Product Moment Correlation coefficients among questionnaire items were positive and moderate to strong. Item-total correlations of .5 were exceeded for all items and sequential removal of items had negligible effect on the Cronbach's alpha coefficient for the unweighted composite score (Table 2).

**Table 2:** Caring for country questionnaire internal consistency, item factor loads and reliability calculations.

Questionnaire item	Item-total correlation	Item-rest correlation	Alpha when item removed	Factor loading	OFCM factor score weight	κ Test-retest	κ Proxy respondent
Time on country	.84	.75	.85	.87	.14	.88***	.57***
Burning	.86	.78	.84	.86	.27	.76***	.47***
Using country	.86	.79	.85	.89	.19	.81***	.48***
Protecting country	.83	.75	.85	.73	.11	.64***	.37***
Ceremony	.73	.60	.88	.58	.01	.52***	.34***
Artefact production	.65	.50	.89	.50	.05	.90***	.47***
Summed raw score (Range: 6–24)						.89***	.59***

Note: *p < .05, **p < .01, ***p < .001: observed agreement is significantly more than expected agreement

Spearman correlations among the responses to time on country and responses to 'time spent on homelands' (-.77, *p *< .001) and 'primary place of residence in the last year' (.75, *p *< .001) indicated satisfactory concurrent validity. All test-retest quadratic weighted kappa scores exceeded .5, indicating satisfactory reliability. Proxy respondent quadratic weighted kappa scores were lower with only the total score and time on country item exceeding .5. However, for both individual and proxy respondent completion, observed agreement always significantly exceeded expected agreement (*p *< .001) (Table 2).

**Figure 1:**
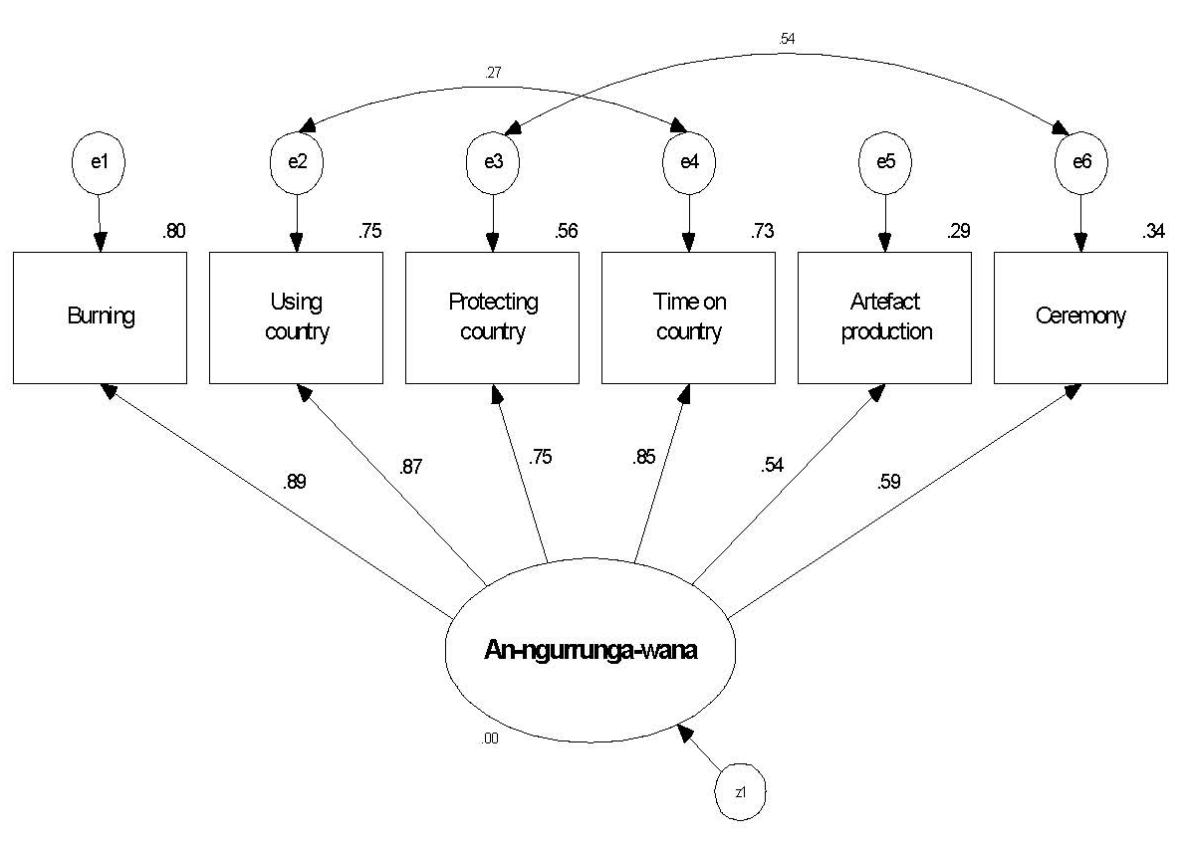
**Fitted one factor congeneric model and standardised estimates of *an-ngurrngna-wana***.

Exploratory factor analysis of the caring for country items generated a one-factor solution accounting for 63.6% of variance in *an-ngurrnga-wana*, with all factor loadings exceeding the criterion of .45 (Table 2). As this was a single factor solution, rotation was not appropriate. The initial one-factor congeneric model (OFCM) did not fit the data well. As there were no non-significant variables or loadings, no items or pathways were deleted and the modification indices were inspected. Two pairs of error terms, concurring with Reid's land, body and spirit domains, were covaried one at a time. The model was comprehensively re-evaluated after each covariance. The final model fitted the data well and achieved the lowest value for the parsimony indices, indicating the model had not been over-fitted (Figure 1 and Table 3). Questionnaire items were multiplied by the factor weights obtained from the OFCM and then summed to generate weighted caring for country scale scores.

**Table 3:** Fit indices for one-factor congeneric model – unfitted and fitted models.

Fit Index	Acceptable values	Unfitted model	Fitted model
CMIN	p > .05	56.69	16.82*
CMIN/DF	1 to 2	6.30	2.40
RMSEA	< .08	.13	.07*
RMR	< .05	.11	.046*
GFI	> .90	.96*	.99*
AGFI	> .90	.91*	.97*
TLI	> .90	.83	.95*
CFI	> .95	.90	.98*
NFI	> .95	.88	.97*
AIC	Lowest	80.69	44.82*
**CAIC**	Lowest	137.17	110.72*

Note: * = acceptable value

All caring for country items were negatively correlated with BMI such that mean BMI decreased as mean caring for country scores increased (Table 1). Four items produced significant zero-order correlations and five items significant partial correlations, controlling for age, gender and residence (Table 4).

**Table 4:** Pearson Product Moment Correlation coefficients among caring for country questionnaire items, and zero-order and partial correlations with body mass index.

	2	3	4	5	6	Body mass index	
						
						Zero-order	Partial^1^
1. Time on Country	.80***	.77***	.58***	.43***	.39***	-.12*	-.12*
2. Using Country	--	.76***	.64***	.48***	.40***	-.18**	-.20**
3. Burning		--	.60***	.46***	.47***	-.15**	-.19**
4. Protecting country			--	.72***	.43***	-.15**	-.19**
5. Ceremony				--	.39***	-.04	-.05
6. Artefact production					--	-.06	-.12*
Weighted scale score						-.17**	-.22***
Index						-.12*	-.15**

Notes: 1. Controlling for gender, age, residence and shared associations with the other caring for country items.

* *p *< .05, ** *p *< .01, ***p < .001.

Zero-order and partial correlation between each item and BMI were larger and stronger for the weighted scale than for the index; the weighted scale score was used in all further analyses.

### Caring for country and health behaviours

Multivariate relationships between social determinants, residence, health behaviours and the caring for country weighted composite scale score were tested using logistic regression (smoking and alcohol) or ordinal logistic regression (diet and physical activity) predicting heath behaviours. Socio-demographic variables (age, gender, income, education and residence) were included in the first step and other health behaviours (smoking, alcohol, takeaway, store foods, physical activity and caring for country) in the second step. Bush food consumption was excluded from the modelling because this was part of the construct definition of caring for country. Non-significant predictors of each health behaviour were eliminated at each step, one at a time, starting with the variable with smallest beta-value. The model was comprehensively re-evaluated after each deletion until only significant predictors remained.

Age and alcohol significantly and independently predicted smoking, with alcohol consumption associated with a threefold likelihood of smoking (Table 5). Being male and smoking predicted alcohol use with smoking associated with an almost fourfold likelihood of drinking. Being male, greater education, living in a homeland and caring for country independently predicted greater physical activity with caring for country demonstrating the strongest independent association. Being female, older age, greater education, homelands residence and caring for country each independently predicted less frequent consumption of takeaway food, with residence and caring for country displaying around a two-fold reduction. Being female and, in particular, store vegetable consumption, were associated with more frequent consumption of store fruit, while age and greater physical activity independently predicted less frequent consumption. Greater income and especially consumption of store fruit were independently associated with more frequent consumption of store vegetables. Being female, caring for country, alcohol use, higher education levels, and greater physical activity were independently associated with more frequent bush meat consumption while consumption of takeaway was associated with less frequent bush meat consumption. Homelands residence, being female, higher education level, alcohol use and caring for country were independently associated with more frequent bush fruit and vegetable consumption. Being a homelands resident was the most significant predictor for greater frequency of bush food consumption.

**Table 5:** Logistic and ordinal logistic regression estimates for the prediction of health behaviours by socio-demographic factors, residence, health behaviours and weighted composite caring for country score.

	Odds ratio	S E	*95% CI*	*Pseudo R^2^*
*Smoker*				.12***
Age	1.07***	.02	1.04 – 1.10	
Alcohol use	3.36*	1.27	1.61 – 7.04	
				
*Alcohol use*				.08***
Female gender	.29***	.08	.17 – .52	
Smoker	2.52**	.80	1.35 – 4.73	
				
*Physical activity*				.08***
Female gender	.55**	.12	.35 – .85	
Education level	1.55***	.19	1.22 – 1.97	
Homeland resident	2.26*	.76	1.17 – 4.38	
Caring for country	2.31***	.55	1.45 – 3.68	
				
*Takeaway consumption*				.06***
Age	.97*	.01	.95 – .99	
Female gender	.53*	.13	.32 – .87	
Education level	.72*	.10	.55 – .94	
Homeland resident	.45*	.18	.21 – .99	
Caring for country	.52*	.14	.30 – .90	
				
*Store fruit consumption*				.23***
Age	.97*	.01	.94 – .99	
Female gender	1.80*	.31	1.07 – 3.03	
Store vegetables	14.29***	3.56	8.78 – 23.25	
Physical activity	.70*	.11	.52 – .95	
				
*Store vegetable consumption*				.22***
Income level	1.79*	.49	1.05 – 3.05	
Store fruit consumption	16.29***	3.87	10.23 – 25.95	
				
*Bush meat consumption*				.15***
Female gender	2.31**	.62	1.37 – 3.90	
Homeland resident	4.71***	1.96	2.09 – 10.63	
Education level	1.36*	.18	1.04 – 1.76	
Physical activity	1.38*	.22	1.02 – 1.88	
Takeaway consumption	.65*	.14	.43 – .99	
Alcohol use	1.78*	.50	1.01 – 3.06	
Caring for country	2.15**	.52	1.34 – 3.45	
				
*Bush fruit & vegetable consumption*				.15***
Female gender	4.95***	1.31	2.95 – 8.30	
Homeland resident	5.65***	2.10	2.73 – 11.71	
Education level	1.47**	.19	1.14 – 1.88	
Alcohol use	2.21**	.60	1.31 – 3.75	
Caring for country	2.36***	.58	1.45 – 3.81	

Note: * *p *< .05, ** *p *< .01, ***p < .001.

As gender was a significant independent predictor of six of the eight health behaviours, we tested interaction terms between sex and caring for country, diet, substance use and physical activity predicting BMI. As most of these terms were strongly and significantly associated with BMI, we analysed data for women and men separately.

### Caring for country and BMI

Hierarchical linear regression modelling was used to test caring for country as a predictor of BMI (Table 6). Non-significant predictors were deleted, one by one, starting with the predictor with the lowest beta value, until only significant predictors were retained in the models.

**Table 6:** Multivariate regression estimates for the prediction of BMI by socio-demographic factors, residence, health behaviours and weighted composite caring for country score for men and non-pregnant women.

	B	S E B	*95% CI*	*β*	*R^2^*
Men (N = 177)					
*Step 1: Socio-demographics*					.04**
Age	.10	.04	.03 – .17	.21**	
*Step 2: Health behaviours*					.09***
Age	.13	.04	.06 – .21	.27**	
Smoker	-1.73	.85	-3.41 – -.07	-.16*	
Caring for country	-1.50	.52	-2.53 – -.47	-.21**	
					
Non-pregnant women (N = 119)					
*Step 1: Socio-demographics*					.04*
Age	.12	.06	.01 – .23	.20*	
*Step 2: Health behaviours*					.07**
Age	.13	.06	.04 – .26	.25**	
Caring for country	-2.10	.87	-3.83 – -.37	-.22*	

Note: * *p *< .05, ** *p *< .01, ***p < .001.

In the first step, for both men and non-pregnant women, only age made a significant independent contribution to explaining BMI scores, accounting for 4% of the variance. In the final model for men, in order of importance, age, caring for country, and smoking were independently related to BMI, accounting for 9% of variance in men's BMI. For non-pregnant women, in order of importance, age and caring for country were independently related to BMI, accounting for 7% of variance in women's BMI.

Given mean heights of 1.60 m and 1.71 m for non-pregnant women and men respectively, and a caring for country weighted scale inter-quartile range of 1.14 and 1.23, non-pregnant women who participated in caring for country activities weighed, on average, 6.1 Kg (95% CI = 1.1–11.2) less than non-participants and men 5.3 Kg (95% CI = 1.6–9) less.

## Discussion

We have demonstrated the preliminary validity of an inductively derived questionnaire measuring Indigenous participation in caring for country activities and described a significant and substantial inverse association with BMI. We found that participation in caring for country activities was significantly associated with greater physical activity, less frequent consumption of takeaway and more frequent consumption of bush foods – health behaviours that contribute to less obesity [29]. These findings are consistent with previous research documenting the health benefits of homelands residence [8] and reinvigoration of a 'traditional lifestyle' [29].

Consistent with recent findings in a comparable remote Indigenous community [49], mean BMI levels in this study indicate a lean population compared to Australia's national prevalence of 51% of overweight and obesity in adults (defined as BMI ≥ 25) [50]. However, this does not imply less risk for development of diabetes and cardiovascular disease, because these diseases occur at lower BMI levels among Indigenous people, with risk increasing incrementally with rising BMI [33,34,49,51].

Male and female participants reported different health behaviours, but similar associations with BMI. For men, health behaviours were associated with BMI as hypothesised. Unexpectedly, given the similar prevalence of smoking for men and non-pregnant women, for women, smoking did not demonstrate an independent relationship with BMI. This may be due to fewer numbers of cigarettes smoked each day (not measured in this study) but this requires examination in further work.

We investigated the reliability and validity of the questionnaire in a challenging setting and it demonstrated satisfactory internal consistency. Reliability was demonstrated through acceptable test-retest and proxy respondent agreement [52]. Content validity was achieved through a two-year collaboration with key Indigenous and non-Indigenous informants from within the study setting. We could not pit our measure against an existing gold standard measure of Indigenous Caring for Country because no such measure is available; indeed, a strength of our research is the development of such a measure. In demonstrating moderate agreement among additional items assessing time on country, it shows concurrent validity. Construct validity was demonstrated by (i) exploratory factor analysis indicating a one-factor solution, consistent with the local Indigenous construct of *an-ngurrunga-wana*, (ii) a fitted one factor congeneric model, also consistent with our hypothesis and with the Yolngu health tri-partite of land, body and spirit, (iii) higher caring for country scores among expected groups, such as homelands residents, and (iv) the significant and substantial association of the scale score, in the expected directions, with key health behaviours and with our external reference, obesity.

The weighted scale score achieved stronger and more substantial associations with BMI than did the index, indicating that total quantity of participation is more important in achieving a lower BMI than is breadth of participation. This is consistent with the proposition that greater physical activity and a healthier diet, associated with caring for country practices, would deliver a more favourable metabolic state [29]. However, breadth of participation in caring for country activities may be important in other socially mediated outcomes, as it is for mental health [45]. This requires further research.

We observed a strong association in this study between residence in homelands and greater participation in caring for country. Residence also demonstrated significant independent associations with less frequent takeaway consumption, more frequent physical activity and more frequent consumption of bush foods – behaviours that would be expected to contribute to a lower BMI [29,53]. Unexpectedly, however, residence was not a significant independent predictor of BMI in the final regression model. This may indicate that participation in caring for country activities mediates the relationship between residence and weight, suggesting that homeland residence is associated with lower weight *because *it engenders a healthier lifestyle. If so, this highlights the value of adequately resourced programs that support Indigenous ranger groups (predominantly based in township locations) and residents in homelands, both of whom maintain caring for country practices [23,24].

### Limitations of this study

We present four main limitations in this study. Firstly, as ours is a cross-sectional study, we are unable to determine the causal direction between caring for country and BMI. However, this was not an aim of our study, which was, instead, to validate a measure of an Indigenous asserted health promotion activity and to relate it to an external reference, obesity. Consistent with a longitudinal study of homelands residents in central Australia that observed significantly lower BMI over time, compared to township residents [8], our findings indicate that caring for country is associated with health behaviours that are likely to impede weight gain. Given the strength of our findings, a longitudinal study is merited.

Second, there may be a selection bias in this study. Volunteers for a preventive health check may not be representative of the population burden of morbidity as they tend to be more health-conscious [54]. Additionally, those with established disease and receiving treatment may be less likely to participate. However, we purposively sampled just under a quarter of the eligible population, aged 15 – 54 years. This sample did not differ significantly from the census age profile [4]. We also achieved a high questionnaire response rate. Further, if those with established disease or poor health were excluded, the results of this study would constitute (i) a conservative estimate of the health benefits of caring for country and (ii) increased probability that caring for country is linked to better health because those physically unable to care for country were excluded from this study. Finally, while a stratified random sample may have been a desirable alternative sampling strategy, this was impractical in the research setting due to (i) high population mobility, (ii) the absence of an accurate community population list and (iii) the need to obtain a much larger sample and collect data for a wider range of co-morbidities.

Third, several of our measures were crude, reliant on self-report and administered in English. Other measures were Eurocentric; for example, income did not include all forms of subsistence production [25], and education did not include traditional knowledge, which is equally important in Indigenous communities [41]. While some self-reported health behaviours, such as dietary assessment, are notoriously inaccurate [55], we could not undertake objective measurement of all behaviours subject to the questionnaire items because participants were widely dispersed and may have found it intrusive. We expect this issue to arise for other research teams. To address it, we have tested the reproducibility of self-reported caring for country activities through test-retest, triangulation with proxy respondent rating and sophisticated statistical modelling. Our methods are consistent with and extend previous research in remote Indigenous communities that have used respondent rating to investigate health behaviours [36]. Unfortunately, translation was not possible due to the lack of qualified interpreters for the eleven language groups. Nevertheless, our questions, piloted and refined with Indigenous health workers in preparation for the study, were considered comprehensible and in a suitable format for this population. Plausible associations between caring for country and health behaviours and obesity support this assessment.

Finally, this scale was developed in a single remote Indigenous community in Arnhem Land fifty years after the founding of the township. Other Indigenous communities with differing linguistic and cultural heritage, or even this community in coming years, may define caring for country differently. However, much of the ethnographic theory underpinning our scale development came from other NT Indigenous communities with longer periods cultural disruption [18,19]. More broadly, the cultural expression, protection of the environment, healthier lifestyles and participation in society encompassed by the questionnaire items resonate with a Maori model of health promotion [56] and the health concepts outlined in the Geneva convention on the health and survival of Indigenous peoples [57].

We argue that the caring for country activities would be relevant to other remote Indigenous populations on their own land in remote areas of Australia. Indigenous Australians possess great diversity in linguistic and cultural traditions and the questionnaire requires further testing in different settings. However, the study cohort was generally representative of remote NT Indigenous peoples in terms of language diversity, residence patterns and a varied participation in customary and contemporary caring for country activities. Further studies are required in similar communities to test the generalisability of this questionnaire and investigate associations between caring for country and other health outcomes.

## Conclusion

We have used a theory-based cross-sectional study to systematically validate an Indigenous-specific questionnaire for participation in caring for country activities and its relationship to a health outcome and health behaviours relevant to premature Indigenous morbidity and mortality. The questionnaire performed well in this cohort across all tests. Further work investigating associations with a broader array of health outcomes is ongoing.

Formal Indigenous caring for country programs have received some support to date [58,59], but are largely reliant on income support payments [23]. We believe that a substantial expansion of investment in Indigenous management of their lands to perform essential environmental services would reap significant health benefits in addition to known environmental benefits [23,60]. This could be relatively inexpensive, low-risk and easy to implement, yet would deliver ecological gains [61], economic development, for example through participation in emerging carbon trading markets [62] and, potentially, health gains via physical, social and cultural pathways [32].

Our study provides empirical epidemiological support for long-standing Indigenous demands for institutional investment in managing their country [24]: such investment could have substantial health and cultural benefits for Australia's most disadvantaged and dispossessed peoples. We emphasise the importance of engaging with Indigenous-asserted health promotion activities that could well make a contribution – inexpensively and respectfully – to tackling seemingly intractable disadvantage in remote Australia.

## Competing interests

The authors declare that they have no competing interests.

## Authors' contributions

CPB conceived the study, undertook the data collection, statistical analysis and had primary responsibility for drafting the manuscript. HB assisted with the study design, supervised the statistical analysis, presentation, interpretation of results and critically reviewed the manuscript. WG participated in the design of the study and reviewed the manuscript. RB provided advice on data analysis and interpretation and helped draft the manuscript. All authors read and approved the final manuscript.

## Supplementary Material

Additional file 1 Appendix 1. Caring for Country questionnaire. Click here for file

Additional file 2 Appendix 2. Theoretical dimensions of the Caring for Country questionnaire [38]. Click here for file
