# Supplementary material for: Development and preliminary validation of the 'Caring for Country' questionnaire: measurement of an Indigenous Australian health determinant
Source: Int J Equity Health. 2008 Dec 18;7:26. doi: 10.1186/1475-9276-7-26 (PMC2628914; doi:10.1186/1475-9276-7-26)
Supplement: Additional file 1 — Appendix 1. Caring for Country questionnaire. [file 1475-9276-7-26-S1.doc]

**Appendix 1: Caring for Country questionnaire**

| **In the last year, how much did you do these things?** | **Not much**  **None in the last year** | **A little bit**  **A few days in the last year** | **A fair bit**  **A few weeks in the last year** | **Heaps**  **A few months in the last year** |
| --- | --- | --- | --- | --- |
| **Time on country**  Living in homeland,  Traveling through country |  |  |  |  |
| **Burning Grass**  Cleaning up Country,  Fire work |  |  |  |  |
| **Using Country**  Bush tucker, Bush medicine,  Hunting, Fishing |  |  |  |  |
| **Protecting Country**  Sacred sites, Animals, Totems |  |  |  |  |
| **Ceremony** |  |  |  |  |
| **Making artworks**  Painting, Weaving, Carving |  |  |  |  |
