# Supplementary material for: Development and preliminary validation of the 'Caring for Country' questionnaire: measurement of an Indigenous Australian health determinant
Source: Int J Equity Health. 2008 Dec 18;7:26. doi: 10.1186/1475-9276-7-26 (PMC2628914; doi:10.1186/1475-9276-7-26)
Supplement: Additional file 2 — Appendix 2. Theoretical dimensions of the Caring for Country questionnaire [38]. [file 1475-9276-7-26-S2.doc]

**Appendix 2: Theoretical dimensions of the Caring for Country questionnaire [38].**

**Land**

**Spirit**

**Body**

An-ngurrugna-wana

Burning

Time on Country

Using Country

Artefact production

Ceremony

Protecting Country
